# Prevalence of anemia and associated factors among school children in Gondar town public primary schools, northwest Ethiopia: A school-based cross-sectional study

**DOI:** 10.1371/journal.pone.0190151

**Published:** 2017-12-28

**Authors:** Zegeye Getaneh, Bamlaku Enawgaw, Getabalew Engidaye, Masresha Seyoum, Muruts Berhane, Zegeye Abebe, Fikir Asrie, Mulugeta Melku

**Affiliations:** 1 Department of Hematology and Immunohematology, School of Biomedical and Laboratory Science, College of Medicine and Health Sciences, University of Gondar, Gondar, Ethiopia; 2 Debre Birhan Referral Hospital Laboratory, Debre Birhan, Ethiopia; 3 University of Gondar Hospital Laboratory, Gondar, Ethiopia; 4 Mekelle University Referral Hospital Laboratory, Mekelle, Ethiopia; 5 Department of Human Nutrition, Institute of Public Health, College of Medicine and Health Sciences, University of Gondar, Gondar, Ethiopia; TNO, NETHERLANDS

## Abstract

**Background:**

Anemia is a global public health problem affecting 305 million school children (SC) worldwide. It has deleterious effects in SC, including lower school achievement due to impaired cognitive development and physical growth, fatigue and poor attention span, and increased morbidity because of reduced resistance to infection. Hence, the aim of this study was to assess the prevalence and associated factors of anemia among SC attending public primary schools in Gondar town, northwest Ethiopia.

**Methods:**

A school-based cross sectional study was conducted on a total of 523 SC aged from 6–14 years old. Multi-stage sampling followed by systematic random sampling techniques were employed to select study participants. Data on socio-demographic and socio-economic characteristics, and dietary status of children were collected using pre-tested structured questionnaire through face-to-face interview of children’s caregivers. Anthropometric measurements were taken. Hemoglobin (Hb) concentration was determined by using HemoCue 301**+**analyser. Stool and blood samples were collected in the school premises, and examined for intestinal and hemoparasites, respectively. Data were entered into Epi Info version 3.5.3 and transferred to SPSS version 20 for analysis. Bivariate and multivariate binary logistic regression models were fitted to identify associated factors of anemia. P-value < 0.05 was considered as statistically significant.

**Result:**

Of the total SC participated in the study, 269 (51.4%) were males. The median (inter quartile range (IQR)) age was 12 (10–13) years, and 332 (63.5%) of them were in the age group 11–14 years. About 81 (15.5%; 95%CI: 12.4%, 18.7%) of them were anemic: 56 (69.1%) and 25 (38.9%) of them were mildly and moderately anemic, respectively. Low maternal education (AOR = 2.30; 95%CI: 1.11, 4.78), stunting (AOR = 2.22; 95%CI: 1.30, 3.80), severe food insecurity (AOR = 5.11; 95%CI: 1.53, 17.13), and soil-transmitted helminthic (STH) infection (AOR = 7.13; 95%CI: 3.16, 16.86) were found significantly associated with anemia.

**Conclusion:**

Anemia among SC was found to be a mild public health problem. It was strongly associated with low maternal education, food insecurity, stunting and intestinal helminthic infection. Focused policies and strategies towards the above factors should be designed to reduce anemia among SC.

## Background

Anemia is a global public health problem affecting the majority of the population of the world in both developed and developing countries with major consequences on human health as well as social and economic development. It is the world’s second leading cause of disability of the whole global disease burden [1–3]. It is considered as a public health problem when the hemoglobin (Hb) value is below the population-specific Hb threshold. It can be classified as no, mild, moderate and severe public health problem when the prevalence is ≤4.9%, 5.0–19.9%, 20.0–39.9%, and ≥40%, respectively [4].

Globally, anemia affects around 305 million (25.4%) SC (children aged 5.00–14.99 years) [1], and it is three to four times more prevalent in non-industrialized regions than industrialized ones. The prevalence of anemia in SC ranges from 5% in North America to 22% in Europe and 30–63% in Asia [1, 5–7]. It also affects millions of SAC in resource-limited countries, especially in Africa and Asia. For example, a study conducted in eight countries of Africa and Asia showed that 12–58% of SAC were suffering from anemia and 12, 41, 54, 57, and 58% in Malawi and Kenya, Ghana, Mozambique, Tanzania, and Mali, respectively, and 30% in Vietnam and Indonesia [8].

According to the 2011 Ethiopia Federal Ministry of Health report on the assessment of the feasibility and potential benefits of food micronutrient fortification, anemia prevalence was estimated to be 24% among children less than 15 years old [9].

In SC, factors contributing to the high prevalence of anemia include inadequate consumption of nutrient-rich foods, lack of access to health care service, and inefficient utilization of available micronutrients as a result of infectious diseases, particularly malaria and helminthic infections [10–13]. Low maternal educational status, poor nutritional practices and unhealthy food habits are additional factors for the development of anemia in SC [12].

The consequences of anemia in SC are unfavourable and adversely affects their cognitive performance and motor development leading to fatigue, low productivity, reduced work capacity, low cognitive function, retarded physical growth, impaired school performance, poor coordination of language and motor skills, and a 5 to 10 points intelligent quotient deficit [4, 7, 14–16].

Since 2013, the Ethiopian Government has been working to address the nutritional concern of SAC, mainly by integrating nutritionally sensitive interventions, such as school biannual deworming and Water Hygiene and Sanitation (WASH) programs in schools [17]. The implementation strategies to prevent and treat anemia are tailored to local conditions which take the specific etiology and prevalence of anemia in a given setting into account. Although it was indicated on National Nutrition Program-II that the evaluation of anemia as part of nutritional assessment has to be conducted at schools, there have been limited national representative findings on the magnitude and factors contributing for the development of anemia in SC, particularly in the study area. Therefore, this study was designed to explore the magnitude of anemia and identify associated factors among SC attending public primary schools in Gondar town, northwest Ethiopia.

## Materials and methods

### Study setting, population, and sampling techniques

A school based cross-sectional study was conducted among SC aged 6–14 years attending public primary schools in Gondar town, 727 kilometres northwest of Addis Ababa, the capital city of Ethiopia, from February to May, 2017. The town is situated at a latitude and longitude of 12°36′N and 37°28′E, respectively, with an elevation of 2133 meters above sea level. According to the 2007 population and housing census of Ethiopia, the projected total population of Gondar town in 2015 was 323,900 [18]. According to the Education Office of the town, there were 15,175 SC attending 26 public primary schools in the 2016/2017 academic year. The sample size (542) was calculated using a single population proportion formula by considering a 30.9% prevalence of anemia among SC [11], 5% margin of error, 95% confidence interval, 1.5 design effect, and a 10% non-response rate. Multi-stage sampling followed by systematic random sampling technique were used to select the study participants. First, all public primary schools in the town were registered, and seven were randomly selected, using the lottery method. Second, the study participants in the target age group enrolled in the selected schools were identified, and the total number of students was obtained from each school director. The sample size was distributed among the selected schools proportionally based on student size in each school. Then, the number of participants required to be enrolled were allocated proportionally based on the number of students in each class and grade level. The K value was calculated by dividing the total number of students in each section by the number of children included in the study. The first student was selected randomly between one and K using, the lottery method, and the next students were selected in accordance with K values until the sample size allocated to each school was reached. Finally, the systematic random sampling was used to select participants. After the students had been identified, their parents were contacted through directors, children and health extension workers.

### Data collection tools and methods

The data collection tools were prepared based on the national survey questionnaire and accordingly modified based on the reviewed literature [19]. The questionnaire design included the following four parts. The first part dealt with the socio-demographic characteristics, like home environment and household items of the child and respective parents/guardians. The second part was designed by incorporating questions on household food security status (HFSS) and feeding practices. Household food security was assessed by using the standardized questionnaire developed by Food and Nutritional Technical Assistance (FANTA) [20]. Part three was designed to determine child feeding practices. A modified version of the Helen Keller International Food Frequency Questionnaire (FFQ) and a 24-hour dietary recall were used to determine food consumption pattern and dietary diversity scores (DDS), respectively [21, 22]. DDS was calculated from ten food groups, and children getting ≥7 food groups were classified as getting high dietary diversity; otherwise, they were considered as getting medium and low dietary diversity score of 4–6, and ≤3, respectively. The fourth part dealt with the morbidity conditions of the children.

#### Socio-demographic and economic data collection

A pre-tested structured questionnaire prepared in English and translated to the local language *(Amharic)* was used to obtain socio-demographic, socio-economic and other related data from the students and parents via face-to-face interviews. The household wealth index was determined using Principal Component Analysis (PCA) by considering household assets and type of house. First, variables were coded between 0 and 1. Secondly, variables were entered and analysed using PCA, and variables with communality values of greater than 0.5 were used to produce factor scores. Finally, factor scores were summed and ranked into tertiles as poor, medium, and rich.

#### Anthropometric measurements

Anthropometric data were collected by recording age, weight, and height of the participants according to WHO guideline [23]. All measurements were done in the school premises. A portable weight scale and a locally made stadio-meter with a sliding headpiece were used to measure weight (to the nearest 0.1 kg) and height (to the nearest 0.1 cm), respectively. Each child was weighed with minimum clothing and barefoot. The weighing scale was calibrated using the standard calibration weight of 2 kg iron bars. Height was measured in Frankfurt position (head, shoulder, buttocks, knee, and heals touch the vertical board) to the nearest 0.1 cm using a locally manufactured wooden standiometres with a sliding head bar. Measurements of weight and height were taken twice and the average was recorded. Then anthropometric measurements were converted into Height-for-Age Z scores (HAZ) and Body Mass Index (BMI)-for-Age Z scores (BAZ) using WHO Anthro Plus (version 1.0.4). Children whose BAZ below -2 standard deviation (SD) of the WHO standard were classified as wasted. On the other hand, stunting was used as an indicator of chronic malnutrition and defined as HAZ <-2 SD of the WHO standard [24].

#### Hemoglobin determination

The Hb concentration of each participant was measured by taking a finger-prick blood sample using a portable hemoglobinometer instrument (HemoCue 301+, Angelholm, Sweden) which is recommended by WHO for the use of field surveys [25] by two trained senior laboratory technologists. Hb readings were corrected for altitude by subtracting the expected normal increase in altitude proposed by WHO based on the altitude of the town nearest to each school from the measured value. Hb concentration adjusted to sea level was used to define anemia. Anemia was defined as; Hb <11.5 g/dl for SC aged 6–11 years, and Hb < 12 g/dl for children aged 12–14 years. The severity of anemia was categorized as mild (Hb between 10 and 11.4 g/dl for 6 to 11 years, and between 10 and 11.9 g/dl for 12 to 14 years), moderate (Hb between 7 and 9.9 g/dl), and severe (Hb < 7 g/dl) for 6 to 14 years of age SC based on the adjusted Hb concentration recommended by the WHO scheme [4].

#### Parasitological examination

From each study participant, fresh stool samples were collected following the standard operating procedures (SOPs) in clean and labelled leak-proof stool cups. The stool specimens were transported in screw-capped cups in 10% formalin to the laboratory. Intestinal helminths were detected microscopically by direct saline wet mount preparation and formol-ether concentration method within 2–8 hours after collection at the University of Gondar, School of Biomedical and Laboratory Sciences Laboratory. Children who were febrile were screened for malaria parasites. Thick and thin blood smear slides were prepared, stained with Giemsa (10%), and examined microscopically for malaria parasites. Malaria was excluded when thick blood films were reported negative after examining 100 fields under x100 oil immersion objective.

### Data analysis and interpretation

Data were entered into Epi Info statistical software version 3.5.3 and analysed using SPSS version 20 software. Data cleaning was performed to check for frequency, accuracy, consistency, and missed values. Any errors identified were corrected. Descriptive statistics (mean, frequency, cross tabulation) were carried out to describe the participants in relation to relevant variables. Bivariate binary logistic regression model was fitted to identify factors associated with anemia. Variables with a p-value of < 0.25 in the bivariate analysis were fitted into the multivariate binary logistic regression analysis. Both crude odds ratio (COR) and adjusted odds ratio (AOR) with the corresponding 95% confidence interval (CI) were calculated to show the strength of association. In the multivariate analysis, variables with a p-values of < 0.05 were considered statistically significant.

### Ethical consideration

The study was conducted after ethical approval was obtained from the University of Gondar, College of Medicine and Health Sciences, School of Biomedical and Laboratory Sciences Research and Ethical Review Committee (Reference number SBMLS/625/09). Permission was taken from Gondar town health and education offices to conduct the study, and a written informed consent was obtained from participants’ parents/guardians. Moreover, assent was obtained from children who were greater than 7 years old. Confidentiality was strictly maintained. All participants who were diagnosed positive for intestinal parasites and anemia were linked to nearby health institutions for standard treatment.

## Results

### Socio-demographic and socioeconomic characteristics of the study participants

A total of 542 SC were selected for the study; however, 12 (2.2%) of the children’s parents did not volunteer to participate in the study, and 7 (1.3%) children refused to give stool and blood samples during sample collection. Five hundred twenty three children with a response rate 96.5% provided complete data (stool and blood specimens and anthropometric measurements) in this study. Of the total 523 SC who participated in the study, 269 (51.4%) were male and 332 (63.5%) were in the age group of 11–14 years. The median (IQR) age of the participants was 12 (10–13) years. Most of the children’s caregivers, 424 (81.1%), were mothers with the mean age of 35.95 (**±**9.632) years. About 230 (44.0%) of mothers had no formal education. The majority of the participants, 414 (79.2%), were categorized in food secured households (Table 1). A total of 100 children who were anemic and febrile were screened for malaria parasite; however, none of them had positive results.

**Table 1:** Socio-demographic and other selected characteristics of SC and their parents, attending at public primary schools in Gondar town, February to May 2017(N = 523).

Variable	Category	Frequency	Percentage
**Age (Child)**	6–10 years	191	36.5
	11–14 years	332	63.5
**Grade level (Child)**	1–4	261	49.9
	5–8	262	50.1
**Family size**	<6	322	61.6
	< = 6	201	38.4
**Child–Guardian Relation-ship**	Mother	424	81.1
	Father	46	8.8
	Relatives	48	9.2
	Others	5	1.0
**Marital status of Mother**	Unmarried	20	3.8
	Married or living together	379	72.5
	separated^**#**^	124	23.7
**Maternal or Guardian age**	< = 34	259	49.5
	35–44	171	32.7
	> = 45	93	17.8
**Mother’s Education**	No formal education	230	44.0
	Primary	160	30.6
	Secondary and above	133	25.4
**Mother’s occupation**	Housewife	364	69.6
	Government employee	51	9.8
	Private*	108	20.7
**Father’s education**	No formal education	214	40.9
	Primary	144	27.5
	Secondary and above	165	31.5
**Father’s Occupation**	Farmer	73	14.0
	Daily labourer	197	37.7
	Government employee	131	25
	Merchant	122	23.3
**Wealth Index**	Low	173	33.0
	Medium	176	33.7
	High	174	33.3
**HFSS**	Food secured	414	79.2
	Mild food insecure	66	12.6
	Moderate food insecure	29	5.5
	Severe food insecure	14	2.7
**DDS**	<4	233	44.6
	*4–6*	228	43.6
	*> = 7*	62	11.8
**Intestinal parasites**	Yes	132	25.2
	No	391	74.8
**STH**	Yes	18	3.4
	No	505	96.6
**HAZ**	Yes	241	46.1
	No	282	53.9
**BAZ**	Yes	46	8.8
	No	477	91.2

Private*: self-employed, selling local drinks, daily labourer

Separated^#^: divorced or widowed

**BAZ:** Body Mass Index**—**for- Age Z score; **HA**Z: Height-for-Age Z score; **DDS**: Dietary Diversity Score; **HFSS**: Household Food Security Status; **STH**: Soil Transmitted Helminths

### Prevalence of anemia

The overall prevalence of anemia among SC was 81 (15.5%; 95%CI: 12.4%, 18.7%), with no statistically significant difference between male (16.0%; n = 43) and female (15.0%; n = 38) (P>0.05). The prevalence of anemia among children in the 6–10 years age group was 16.2% (n = 31), while it was 15.1% (n = 50) among children in the 11–14 years age group. The Hb level of the participants ranged from 9.7 g/dl to 16.3 g/dl with a mean (± SD) value of 12.72 ±1.08g/dl. Of the anemic children, 56 (69.1%) and 25 (30.9%) were mildly and moderately anemic, respectively. However, there was no severe anemia case in this study. The prevalence of anemia was found to be 21.8% (n = 27) and 19.6% (n = 45) among children whose caregivers were not living together and had no formal education, respectively. The prevalence of anemia was higher among SC who were living in food insecure household, 34.6% (n = 28) (Table 2).

**Table 2:** Bivariate and multivariate logistic regression analysis of factors associated with anemia among SC in Gondar town, northwest Ethiopia, February to May 2017 (n = 523).

Variable		Anemia		COR (95% CI)	AOR (95%CI)
		Anemic	Non-Anemic		
**Child’s grade**	Grade 1–4	46 (17.6%)	215 (82.4%)	1.38 (0.86, 2.24)	1.27 (0.72, 2.21)
**Label**	Grade 5–8	35 (13.4%)	227(86.6%)	1.00	1.00
**Age of mothers**	< = 34	227(87.6%)	227(87.6%)	1.00	1.00
	35–44	141 (82.5%)	141(82.5%)	1.51 (0.88, 2.59)	1.37 (0.74, 2.56)
	> = 45	74 (79.6%)	74 (79.6%)	1.82 (0.98, 3.40)	1.81 (0.88, 3.71)
**Marital status**	Married/living together	51 (13.5%)	328 (86.5%)	1.00	1.00
**(care taker)**	Unmarried	3 (15.0%)	17 (85.0%)	1.14 (0.32, 4.01)	1.55 (0.37, 6.47)
	Separated**#**	27 (21.8%)	97 (78.2%)	1.79 (1.07, 3.00)	1.33 (0.71, 2.52)
**Mother’s education**	No formal education	45 (19.6%)	185 (80.4%)	2.07 (1.09, 3.93)	2.37 (1.12, 5.02)*
	Primary	22 (13.8%)	138 (86.2%)	1.36 (0.66, 2.77)	1.81 (0.79, 4.12)
	Secondary & above	14 (10.5%)	119 (89.5%)	1.00	1.00
**Father’s education**	No formal education	39 (18.2%)	175 (81.8%)	1.45 (0.82, 2.56)	1.07 (0.52, 2.22)
	Primary	20 (13.9%)	124 (86.1%)	1.05 (0.55, 2.01)	0.81 (0.37, 1.74)
	Secondary and above	22 (13.3%)	143 (86.7%)	1.00	1.00
**HFSS**	Food secured	53 (12.8%)	361 (87.2%)	1.00	1.00
	Mild Food insecured	15 (22.7%)	51 (77.3%)	2.00 (1.05, 3.81)	2.20 (1.07, 4.50)*
	Moderate food insecured	7 (24.1%)	22 (75.9%)	2.17 (0.88, 5.32)	2.42 (0.85, 6.88)
	Severely food insecured	6 (42.9%)	8 (57.1%)	5.11 (1.71, 15.30)	6.02 (1.84, 19.68)**
**DDS**	<4	50 (21.5%)	183 (78.5%)	2.55 (1.04, 6.26)	1.77 (0.69, 4.55)
	4–6	25 (11.0%)	203 (89.0%)	1.15 (0.45, 2.94)	0.79 (0.29, 2.13)
	> = 7	6 (9.7%)	56 (90.3%)	1.00	1.00
**STH infection**	**Yes**	11 (61.0%)	7 (39.0%)	9.76 (3.66, 26.04)	14.23 (4.80,42.20)**
	No	70 (13.9%)	435 (86.1%)	1.00	1.00
**Coffee-drinking**	Yes	28 (21.1%)	105 (78.9%)	1.70 (1.021, 2.82)	1.25 (0.68, 2.30)
**Habit**	No	53 (13.6%)	337 (86.4%)	1.00	1.00
**BAZ**	Wasted	14 (29.8%)	33 (70.2%)	2.59 (1.32, 5.09)	0.70 (0.25, 1.91)
	Not wasted	67 (14.1%)	409 (85.9%)	1.00	1.00
**HAZ**	Stunted	50 (20.7%)	191 (79.3%)	2.12 (1.30, 3.45)	2.083 (1.22, 3.56)**
Not stunted		31 (11.0%)	251(89.0%)	1.00	1.00

Separated**#**: divorced or widowed

*P-value <0.05

**P-value <0.01

**AOR:** Adjusted Odds Ratio; **COR:** Crude Odds Ratio

The prevalence of anemia was 20.7% and 29.8% among children who were stunted and wasted, respectively. From a total of 132 (25.2%) SC were infected at least one of the seven species of parasites; *Entamoeba histolytica*, *H*.*nana*, *Giardia lamblia*, *Schistosoma mansoni*, *Ascaris lumbricoides*, *Hookworm* and *Entrobius vermicularis*. These intestinal parasites were categorized as soil-transmitted helminths (Ascaris lumbricoides and Hookworm) and non- soil-transmitted helminths (*Entamoeba histolytica*, *Giardia lamblia*, *H*.*nana*, *Entrobius vermicularis* and *Schistosoma mansoni*) during data analysis. Of those children infected by intestinal parasites, 23 (28.4%) were found anemic and of 18 children infected by STH, 11 (61%) were anemic (Table 2).

### Factors associated with anemia in SC

To determine association, bivariate logistic regression analysis was done. Based on the analysis, maternal education; current marital status, food insecurity, children’s dietary diversity, coffee utilization habit, infection by STH, stunting, and wasting were significantly associated with the prevalence of anemia. However, in the multivariate logistic regression analysis, having mother with no formal education (AOR = 2.37; 95% CI: 1.12, 5.02), living in mildly food insecured households (AOR = 6.02; 95%CI: 1.84, 19.68), living in severely food insecured households (AOR = 2.20; 95%CI: 1.07, 4.50), being infected with STH (AOR = 14.232; 95%CI:4.80, 42.20), and being stunted (AOR = 2.08; 95%CI: 1.22, 3.56) remained significantly associated with anemia among SC (Table 2).

## Discussion

According to WHO, anemia is considered as a public health problem when the prevalence is greater than 5% [26]. However, the magnitude of the problem is defined as mild, moderate, and severe when the prevalence is 5–19.9%, 20–39, 9% and > = 40% [4], respectively. Accordingly, 15.5% prevalence of anemia in SC confirmed the existence of a mild public health problem in the study area. This indicated that considerable number of children in the community were suffering from anemia. Anemia is negatively correlated with educational outcomes, leading to poor learning outcomes, such as loss of concentration in class, impaired academic achievement, and discontinuation of education [27, 28]. So that emphasis should be given to reduce the prevalence of anemia in children.

The prevalence of anemia among SC in this study was comparable with the result of other similar study among SC in Aydın, Turkey (15.7%) [29]. But compared to other local studies in Ethiopia, our finding was higher than the study findings from Mekele (11%) [30], and Addis Ababa (5.83%) [17]. This may be explained by the high prevalence of chronic childhood undernutrition in the study area. According to the Ethiopian 2011 and 2016 DHS reports, the highest prevalence of childhood undernutrition was noted in Amhara Region [31, 32]. So, chronic undernutrition is one of the major contributing factors to nutritional anemia [33].

Moreover, the prevalence seen in this study was higher than those of studies conducted in countries, such as the Siauliai region of Lithuania (10.1%) [27], Serbia (10.8%) [34], Mexico (12%) [35], and Brazil (9.3%) [36]. This discrepancy might be due to the variability of risk factors across different geographic regions, plus lower socioeconomic and nutritional status of SC in this study area than in the other settings. On the other hand, the current finding is lower than previous local reports from Kersa (27.1%) [37], Filtu (23.66%) [38], Jimma (37.6–43.7%) [39, 40], Libo-Kemkem and Fogera districts (30.9%) [11]. The possible explanation for the low prevalence of anemia in this study might be due to differences in the study period and the effect of the biannual deworming program which is an important public health intervention that the government recently implemented to decrease intestinal infection by providing antihelminthics and malnutrition.

The prevalence of anemia in this study was lower than those of studies done in other African countries, such as Kenya (28.8–35.3%) [33, 41], Nigeria (25.0–29.2%) [42, 43], Cape Verde, West Africa (23.8%) [44], Mali (58%), Tanzania (57%), Mozambique (54%), Ghana (41%) [8], Sudan (88.3%) [45] and Egypt (59.3%) [46]. It was also lower than the findings of studies conducted in Asian countries, like Gaza strip, Palastine (35.3%) [47], and western China 34% [48]. The discrepancy may be attributed to the differences in study settings (the latter were carried out in rural areas) and the current deworming intervention program in Ethiopia [17]. In addition, the consumption of iron-rich foods, for example, teff, the iron-rich staple food in Ethiopia might have contributed to the low prevalence of anemia in the study area. Furthermore, malaria has been shown to cause anemia in several studies [13], the fact that there was no case of malaria might be the reason for low prevalence of anemia in this study.

In the current study, maternal education was found significantly associated with anemia in SC. Children whose mothers had no formal education were two times more likely to be anemic compared to children whose mothers had secondary and above level of education. This finding is consistent with the result of a study done in Aydin, Turkey [29] and Himachal Pradesh, India [49]. Maternal educational status plays a major role in child health, nutrition, growth and development. Uneducated mothers may not understand the nutritional requirements of children easily and follow the recommended child feeding practices [50]. In addition, mothers’ lack of education negatively affects the socio-economic status of households which in turn limits food purchasing power. Hence, children’s access to heme iron sources, animal products, is limited [51].

Consistent with the study findings elsewhere [39, 44, 52] children living in a food insecured household had a greater chance of being anemic. Accordingly, the odds of anemia were six and two times higher among SC who were living in severely and mildly food insecure households, respectively, than among food secure ones. This might be due to the fact that cereal based monotone diet with poor quality, quantity, and frequency of feeding is the only option for food insecure households which doesn’t fulfil the micronutrient requirements of children, such as iron, vitamin B12, and folate [53].

In our study, stunting was found significantly associated with anemia among SC. Stunted children were two times more likely to be anemic than non-stunted children. This might be due to the long term effect of low intake of both macro and micronutrients, especially iron, vitamin B12, folate and other minerals and vitamins [14]. This was in line with studies conducted in Kersa, Eastern Ethiopia [37], and Filtu town, southeast Ethiopia [38].

Finally, STH infections were found significantly associated with anemia among SC. Children infected with STHs were fourteen times more likely be anemic compared to their counterparts. This is consistent with reports from Filtu town, southeast Ethiopia [38], and Mekele, northern Ethiopia [30]. This might be due to nutritional competition, RBC destruction and feeding, and loss of appetite caused by worms. These parasites also cause impaired nutrient uptake by a direct damage of the intestinal mucosal wall [5, 14].

The first limitation of this study was the cross-sectional nature of the design in that it did not allow us to observe causality in the relationship between anemia and its associated factors. We didn’t differentiate specific causes of anemia, such as iron, vit.B12, folate, and the RBC morphology was not identified. The other limitation of this study is that subclinical infections other than intestinal parasites and malaria were not assessed among school children, which limits generalizability of the study for possible risk factors.

## Conclusion

In this study, Anemia was found to be a mild public health problem among SC. It was strongly associated with low maternal education, food insecurity, stunting, and STH infections. Hence, focused policies and strategies towards school children should be designed to reduce anemia among food insecure, stunted and low income groups. While designing school based intervention strategies, targeting anemia prevention, and nutritional supplementation is imperative in addition to the existing school based deworming program. Moreover, health education that enhances the knowledge of women about child feeding practices should be given regularly. On top of that, further large scale longitudinal studies using a larger sample size, and including the assessment of all red blood cell indices, red cell morphology, serum micronutrient level, and subclinical infections need to be conducted to identify the cause-effect relationships of anemia with its contributing factors.

## Supporting information

S1 File “English version questionnaire” consists of data collection tool in English language. (DOC)

S2 File “Amharic version questionnaire” consists of data collection tool in local language, Amharic. (DOC)

S3 File “Data set used for analysis” includes data on anemia and associated factors. (XLS)

## Abbreviations

AOR: Adjusted Odds Ratio
BAZ: Body Mass Index–for-Age Z-scores
CI: Confidence Interval
COR: Crude Odds Ratio
DDS: Dietary Diversity Score
FFQ: Food Frequency Questionnaire
HAZ: Height-for-Age Z-scores
Hb: Hemoglobin
HFSS: Household Food Security Status
IQR: Inter Quartile Range
RBC: Red Blood Cell
SC: school children
SD: Standard Deviation
SOP: Standard Operating Procedure
STH: Soil Transmitted Helminths
WHO: World Health Organization
