# Supplementary material for: Prevalence of anemia and associated factors among school children in Gondar town public primary schools, northwest Ethiopia: A school-based cross-sectional study
Source: PLoS One. 2017 Dec 28;12(12):e0190151. doi: 10.1371/journal.pone.0190151 (PMC5746225; doi:10.1371/journal.pone.0190151)
Supplement: S2 File — (DOC) [file pone.0190151.s002.doc]

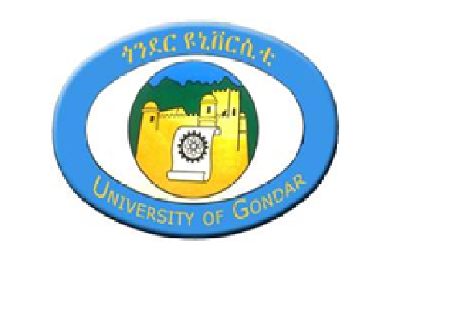
**የጎንደር ዩኒቨርሲቲ የህክምናና ጤና ሳይንስ ኮሌጅ የባዮሜዲካልና ላቦራቶሪ ሳይንስ ትምህርት ቤት** ሄማቶልጂና ኢሚኖሄማቶልጂትምህርት ክፍል

# 1. የመረጃ መስጫ ቅጽ

ስሜ______ይባላል፡፡የመጣሁት ከጎንደር ዩኒቨርሲቲ ህክምናና ጤና ሳይንስ ኮሌጅ የባዮሜዲካልና ላቦራቶሪ ሳይንስ ትምህርት ቤት በክሊኒካል ሄማቶልጂና ኢሚኖሄማቶልጂ ትምህርት ሁለተኛ ዲገሪ ተመራቂ ተማሪ "የደም ማነስ እና ደም ማነስን የሚያስከትሉ አጋላጮችን በተመለከተ ለሚሰራዉ ጥናት የመረጃ ሰብሳቢ ነኝ፡፡ የደም ማነስ በአለም አቀፍ ደረጃ በስፋት ያለ ችግር ሲሆን ከ305 ሚሊዮን በላይ የሚሆኑ እድሜያቸው ለትምህርት የደረሱ ህፃናትን እያጠቃ የሚገኝ በሽታ ነው፡፡ በሀገራችን ደገሞ 24 በመቶ የሚሆኑ እድሜያቸው ለትምህርት የደረሱ ህፃናትን ያጠቃል፡፡ ምንም እንኳን የዚህ በሽታ መንስኤዎች እንደየአካባቢው ሁኔታ የተለያዩ ቢሆኑም የብረት ንጥረ ነገር እጦት/ማነስ አንደኛውና ዋንኛው መንስኤ ነው፡፡ የአንጀት ጥገኛ ተህዋስያን (እንደነ ወስፋት፣ የመንጠቆ ትልና ብልሃርዝያ)፣ ስር የሰደዱ በሽታዎች እንዲሁም የሌሎች ንጥረ ነገሮች እጥረት የደም ማነስ በሽታ አደጋን ሊጨምሩ ይችላሉ፡፡

የጥናቱ አላማም የደም ማነስ በአካባቢው ህጻናት በተለይ እድሜያቸው ከ6 እስከ 14ዓመት የሆኑትን በምን ያህል ደረጃ እያጠቃ እንዳለ ማወቅ ነው ፡፡የእርስዎ ቤትና ልጅ በዚህ ጥናት ተሳታፊ እንዲሆኑ በዕጣ ተመርጣችኋል፡፡ ጥናቱ የህጻናትን የደም እና የሰገራ ምርመራን እንዲሁም ያካላቸውን የድገት ያካትታል፡፡ የደም ማነስ ምርመራ ለማካሄድ ትንሽ የደም ጠብታ ከልጅዎ የግራ እጅ ጣቱ ላይ ከፍተኛ ልምድ ባላቸው ባለሞያዎች በጥንቃቄ እንወስዳለን:: ውጤቱንም ወዲያውኑ እንነግሮታለን፡፡ ምርመራው ህጻኑ ላይ ምንም ችግር እንደማይፈጥር እናረገግጣለን:: ልጅዎ በመመርመሩ የሚያገኘው ጥቅም ደም ማነስ እንዳለበትና እንደሌለበት፤በአንጀት ጥገኛ ትላትል መጠቃት አለመጠቃቱን መረጃ ያገኛሉ በተጨማሪም ከፍተኛ ደም ማነስ ያለባቸውን ወደ ከፍተኛ ህክፍምና ተቋማት ለህክምና እንልካቸዋለን:: እንዲሁም መድሃኒት በአንጀት ጥገኛ ትላትል ለተጠቁ ይሰጣቸዋል፡፡ ማንኛውም የሚሰጡን መረጃ በሚስጢር እንደሚያዝ እናረጋግጣለን፡፡ በዚህ መጠይቅ የሚኖረዎት ተሳትፎ ሙሉ በሙሉ በፈቃደኝነት ላይ የተመሰረተ ነው፡፡ ሆኖም የእርስዎ ልምድና እውቀት ልጆች ላይ የሚከሰተውን የደም ማነስ በሽታ ለመከላከል ከፍተኛ አስተዋፆ ይኖረዋል፡፡ ቃለ ምልልሱና ምርመራው በግምት ግማሽ ሰአት ይፈጃል፡፡

**2. የስምምነት መጠየቅያ ቅጽ**

- ቃለ ምልልስ ለመስጠት ፈቃደኛ ነዎት?

1. ፈቃደኛ ነኝ _____ 2. ፈቃደኛ አይደለሁም_____ አመስግነህ/ሽ ቃለ-መጠይቁን አቁም/ሚ

እባክዎትን ከዚህ በታች የተዘረዘሩ ነጥቦን በጥሞና ያንብቡ እና በመጨረሻ በተሰጠው ክፍት ቦታ ፌርማዎን ያኑሩ::

የደም **ማነስ ሁኔታና ተዛማጅ የሆኑ አጋላጮችን ለማጥናት** የሚካየደውን ጥናት ዓላማውን ተረድቻለሁ። የምሰጠው መረጃ እና ናሙና ለዚህ ጥናት ብቻ እንደሚዉል አውቂያለሁ። ለጥናቱ የምሰጠው መረጃ እና ናሙና እንዲሁም ውጤቱ በሚስጥር እንደሚያዝ ተረድቻለሁ። በጥናቱ በመሳተፌ የሚከፈለኝ ክፍያ እንደሌለ አውቂያለሁ። ስለዚህ ከላይ የተጠቀሱትን ነጥቦች በመረዳት መረጃ ለመስጠት እና ከልጄ የደምና የሰገራ ናሙና እንዲሰጥ ተስማምቻለሁ።

የወላጅ/አሳዳጊ ፊርማ-------------------------- ቀን-------------/---------/-------------

በጠያቂው የሚፈረም፤ ከዚህ በላይ የተመለከተውን መረጃና ስምምነት መፈጸማችን አረጋግጣለሁ ፊርማ-----------------------

**ከ 7-14 አመት ላሉ ልጆች የስምምነት መፈረሚያ ቅጽ**

የሚከተለዉን ቅጽ በማንበብ ከታች በሚገኘዉ የመፈረሚያ ቦታ ይፈርሙ፡፡

በጥናቱ ለመሳተፍ ሙሉ በሙሉ ፈካደኛ ነኝ፡፡ አሳዳጊዎቸም በጥናቱ እንድሳተፍ ፈቀደኛ ሆነዋል፡፡ ስለ ጥናቱ ዓላማ በዋና ተመራማሪዉ/ ተወካዩ በሚገባ ተገልጾልኛል፡፡እንዲሁም ስለጥናቱ መረጃ መስጫ ቅጹን በሚገባ በማንበብ የጥናቱን አላማ ተገንዝቤያለሁ፡፡ የጥናቱ ተመራማሪ ከጣት ላይ ጥቂት ጠብታ የደም እና ሰገራ ናሙና እንደሚወስድም ተረድቻለሁ፡፡ ስለጥናቱ ዓላማና ሂደት ያልገባኝ ነገር ቢኖር እንኳ ተመራማሪዉን/ተወካዩን መጠየቅና መረዳት እንደምችል ተገነዝቤያለሁ፡፡ የተሳትፎየን ዉጤት ማንነቴን በማይገልጽ መልኩ ተመራማሪዉ ለጥናቱ አላማ እንዲያዉለዉ ፈቅጃለሁ፡፡ ተሳትፎየን በማንኛዉም ሰዓት ማቋረጥ እንደምችል ተረድቻለሁ፡፡ ማንኛዉም ከኔ የሚሰበሰበዉ መረጃ በሚስጥር እንደሚያዝ ተገንዝቤያለሁ፡፡

ስም----------------------------------ፊርማ---------------ቀን-------------- የወላጅ/አሳዳጊ -------------------------ፊርማ---------ቀን----------

ቃለ-መጠይቅ

| ክፍል አንድ፡ ሀ. የእናት**/**ተንከባካቢ/አሳዳጊ**ማህበራዊ ና ስነ-ህዝባዊ** ጥያቄዎች  ስለ እናት **/**ተንከባካቢ/አሳዳጊ መረጃ ለመጠየቅ የቀረቡ ጥያቄዎች: አሁን ስለራስዎና ስለ ቤትዎ አስመልክቶ የተወሰኑ ጥያቄዎችን እጠይቆታለሁ | | | | | | | | | | | | | | | | | | | | | |
| --- | --- | --- | --- | --- | --- | --- | --- | --- | --- | --- | --- | --- | --- | --- | --- | --- | --- | --- | --- | --- | --- |
| **መለያ** | ጥያቄዎችና መለያዎች | | መልስና ኮድ | | | | | | | | | | | | | | | ይለፍ | | | |
| 101 | | እርስዎ ከልጁ/ከልጅቷ ጋር ያልዎት ዝመድና? | እናት.......................1 አባት......................2  የእንጅራ አባት……..3 የእንጅራ እናት……4  ወንድም ………….5 እህት………………….6  የቅርብ ዘመድ………7 ሌላ ካለ ይገለጽ……99_____________ | | | | | | | | | | | | | | |  | | | |
| 102 | | የመኖሪያ አድራሻ ሁናቴ ምንድን ነው? | ገጠር…… 1 ከተማ……2 በከፊል ከተማ…..3 | | | | | | | | | | | | | | |  | | | |
| 103 | | እድሜዎት ስንት ነው? | እድሜ__________________ | | | | | | | | | | | | | | |  | | | |
| 104 | | ሀይማኖትዎ ምንድን ነው ? | ኦርቶዶክስ ክርስቲያን............1 እስላም.............................2  ፕሮቴስታንት.......................3 ካቶሊክ................................4  ሌላ ካለ ይገለጥ…………99__________________ | | | | | | | | | | | | | | |  | | | |
| 105 | | እርስዎ ከየትኛው ብሔር ነዎት ? | አማራ..........1 ኦሮሞ.........2 ትግሬ………3 ቅማንት--------4  ሌላ ካለ ግለጥ………99__________________ | | | | | | | | | | | | | | |  | | | |
| 106 | | የእርስዎ የጋብቻ ሁኔታ እንዴት ነው? | ያላገባ/ች--------------1 ያገባ/ች-----------------2  በጋብቻ አብረው የሚኖሩ---------3 በተለያየቦታ የሚኖሩ-------4  የተፋቱ-------------5 ባሏ የሞተባት-------------6 | | | | | | | | | | | | | | |  | | | |
| 107 | | የእርስዎን የትምህርት ደረጃ ቢገልጡልኝ? | አንደኛ ደረጃ(1-8)……1 ሁለተኛ ደረጃ(9-12)…2  ዲፕሎማ/ዩኒቨርሲቲ…..3 ማንበብና መጻፍ የሚችል………4  ያልተማረ…….5 | | | | | | | | | | | | | | |  | | | |
| 108 | | የእርስዎ የስራ አይነት ምንድን ነው?  ***(የልጁ/የልጅቷ እናት /ያሳዳጊ)*** | አርሶ አደርና የቤት እመቤት--------1 የቤት እመቤት-------------------2  የመንግሰት/የግል ተቀጣሪ----------3 አረቄ፣ጠላ መሸጥ--------------4  ነጋዴ--------------5 ተማሪ------6 ሴተኛአዳሪ------------------7  የቤት ሰራተኛ------8 የቀን ሰራተኛ-----9 ስራ የሌለው--------------10  ሌላ ካለ ይገለጽ------99 | | | | | | | | | | | | | | |  | | | |
| 109 | | የልጁ/የልጅቷ አባት የትምህርት ደረጃ ስንት ነው?  ***የልጁ/የልጅቷ አባት (ያሳዳጊ)*** | አንደኛ ደረጃ(1-8)………………1 ለተኛ ደረጃ(9-12)…………….2  ዲፕሎማ/ዩኒቨርሲቲ …………….3 መንበብና መጻፍ………………4  ልተማረ………………………5 | | | | | | | | | | | | | | |  | | | |
| 110 | | የልጁ/የልጅቷ አባት የስራ አይነት ምንድን ነው? | አርሶ አደር…………………1 የመንግስት/የግል ተቀጣሪ……2  ተማሪ……3 ነጋዴ…4 የቀን ሰራተኛ………………..5 ስራ የሌለው…………6 ሌላ ካለ ይገለጽ……………99 | | | | | | | | | | | | | | |  | | | |
| 111 | | ጠቅላላ የቤተሰብ አባል ስንት ነው? | ------------------------------------- | | | | | | | | | | | | | | |  | | | |
| ለ. የመልስ ሰጪውን ቤትና ቤት ውስጥ ያለውን ንብረትና መረጃ በተመለከተ  ለመረጃ ሰብሳቢ**:** ተሳታፊዉ እነዚህን ጥያቄዎችን መመለስ ከከበደዉ/ዳት ሌላ የቤቱን አባል ጠይቆ አስፍላጊዉን መረጃ ማግኘት ይችላሉ፡፡ | | | | | | | | | | | | | | | | | | | | | |
| 112 | | የመኖርያ ቤት ይዞታ | የግል………..1 የቀበሌ……..2 ኪራይ………3 የዘመድ/የሌላ ሰዉ………4  ላሊ (ግለፅ)________________________________ | | | | | | | | | | | | | | |  | | | |
| 113 | | የመኖርያ ቤቱ ግዴግዳዉ የተሰራበት ቁስ | እንጨት ና ጭቃ………1 ብሎኬት………2 ቆርቆሮ……………..3  ዘንባባ/ሸንበቆ/ቀርከሃ/ሣር……..4 ጡብ……5  ዴንጋይ ና ጭቃ/ስሚንቶ…….……6 ሌ(ይጥቀስ)_____________________ | | | | | | | | | | | | | | |  | | | |
| 114 | | የመኖርያ ቤትዎ ምን አይነት የጣራ ክዳን ነዉ ያለዉ?  **(**ለመረጃ ሰብሳቢ፡ ተመልክተህ/ሽ ኣንዱን ኣክብ/ቢ፡፡ክዳኑ የተሰራበት ከኣንድ በላይ በሆኑ አማራጮች ከሆነ አብዛኛዉን የሸፈንበትን ምረጥ/ጪ) | ቀርከሃ/ሸንበቆ/ሣር…………..1 ቆርቆሮ……..2 ጣዉሊ………………3 ፕሊስቲክ………………4 ስሚንቶ/አምነበረድ………….5  ሌላ (ይጥቀስ)_______________________________ | | | | | | | | | | | | | | |  | | | |
| 115 | | የመኖርያ ቤቱ ወለል የተሰራበት ቁስ  (ለመረጃ ሰብሳቢ፡የቤቱ ወለል ሙሉ በሙሉ በምንጣፍ የተሸፈነ ከሆነ ምንጣፍ/ስጋጃ የሚለዉን ምረጫ /ኣክብብ/ቢ) | አፈር/አሸዋ…………1 ጭቃ ................ 2 ጣዉላ .............3  ዘንባባ/ሸንበቆ/ቀርከሃ/ሣር ............ 4 ቀለም የተቀባ የወለል ጣዉላ....…5  ስሚንቶ............... 6 ሴራምክስ ...... ..7 ምንጣ/ስጋጃ………………8  ሌላ (ጥቀሱ)_______________________________ | | | | | | | | | | | | | | |  | | | |
| 116 | | ስንት ክፍሎችን ለመኝታ ትጠቀማላችሁ? | የክፍሎች ቁጥር----------/-------- | | | | | | | | | | | | | | |  | | | |
| 117 | | ከዚህ በታች የተዘረዘሩ ና የሚያገለግል የቤት ቁሳቁስ አለዎት**?** | **አለ--1** | | | | | | | | | | **የለም--2** | | | | |  | | | |
|  |  | ኤሌክትሪክ | **1** | | | | | | | | | | **2** | | | | |  |  |  |  |
|  |  | የግዴግዳ ሰዓት | **1** | | | | | | | | | | **2** | | | | | |  | | |
|  |  | ሬዲዮ | **1** | | | | | | | | | | **2** | | | | | |  |  |  |
|  |  | ቴሌቭዢን | **1** | | | | | | | | | | **2** | | | | | |  |  |  |
|  |  | ተንቀሳቃሽ ስልክ | **1** | | | | | | | | | | **2** | | | | | |  |  |  |
|  |  | የቤት ስልክ | **1** | | | | | | | | | | **2** | | | | | |  |  |  |
|  |  | ማቀስቀዣ ማሽን /ፍሪጅ | **1** | | | | | | | | | | **2** | | | | | |  |  |  |
|  |  | ጠረጴዛ | **1** | | | | | | | | | | **2** | | | | | |  |  |  |
|  |  | ወንብር | **1** | | | | | | | | | | **2** | | | | | |  |  |  |
|  |  | የእስፖንጅ/ጥጥ ፍራሽ ያለዉ አልጋ | **1** | | | | | | | | | | **2** | | | | | |  |  |  |
|  |  | የኤሌክትሪክ ምጣድ | **1** | | | | | | | | | | **2** | | | | | |  |  |  |
|  |  | የጋዝ መብራት | **1** | | | | | | | | | | **2** | | | | | |  |  |  |
|  |  | በፀሃይ ሀይል የሚሰራ መብራት/ሶላር | **1** | | | | | | | | | | **2** | | | | | |  |  |  |
| 118 | | ቤተስብዎ ለምግብ ማብሰያነት በዋናነት ምን የሃይል ምንጭ ይጠቀማል? | ኮረንቲ………………1 የተፈጥሮ ጋዝ…………2  ባዮ ጋዝ…………...3 ነጭ ናፍጣ……………….4  የማገዶ እንጨት……5 ከሰል…………………..6  ገለባ………………..7 ኩበት…………………8  የሰብል አገዳ………9 ሌላካለ ይገለጥ……………99 | | | | | | | | | | | | | | | |  | | |
| 119 | | ከቤተሰቦት አባል የሚከተሉትን ያለዉ ኣለ**?** | አዎ አለ | | | | | | የለም | | | | | | | | | |  | | |
|  |  | ብስክሌት | 1 | | | | | | 2 | | | | | | | | | |  |  |  |
|  |  | ሞተርሳይክል/ዶቅዶቄ | 1 | | | | | | 2 | | | | | | | | | |  |  |  |
|  |  | በእንሰሳ የሚሳብ ጋሪ | 1 | | | | | | 2 | | | | | | | | | |  |  |  |
|  |  | መኪና | 1 | | | | | | 2 | | | | | | | | | |  |  |  |
| **ክፍል ሁለት፡ በቤት ውስጥ የተከሰተ የምግብ እጥረትን በተመለከተ** | | | | | | | | | | | | | | | | | | | | | |
| 201 | | ባለፈው አንድ ወር እርስዎ ወይንም ሌላ የቤተሰብዎ አባል በቤትዎ በቂ ምግብ አኖርም ብለው ሰግተው/ተጨንቀው ያውቀሉ? | | | | አዎ .....................................................1  የለም .................................................0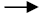 | | | | | | | | | | | | **203** | | | |
| 202 | | ይህ ችግር ምን ያህል ግዜ ተከስቷል? | | | | ***አልፎ አልፎ***(1- 2 ግዜ በወር ዉስጥ) ...1  ***አንዳንዴ***(ከ3-10 ግዜ በወር ዉስጥ)......2  ***ብዙጊዜ*** (ከ10 ግዜ በላይ በወር ዉስጥ)...3 | | | | | | | | | | | |  | | | |
| 203 | | ባለፈው አንድ ወር እርስዎ ወይንም ሌላ የቤተሰብዎ አባል አቅም በማጣት ምክንያት የፈለጉትን የምግብ አይነት መመገብ ሳይችሉ ቀርተዋል? *ለጠያቂ፡ ለምሳሌ ምስር መብላት ፈልገው አልበሉም* ? | | | | አዎ ...................................................1  የለም ................................................ 0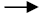 | | | | | | | | | | | | **205** | | | |
| 204 | | ባለፈው አንድ ወር ውስጥ ይህ ችግር ምን ያህል ግዜ ተከስቷል? | | | | ***አልፎ አልፎ***(1- 2 ግዜ በወር ዉስጥ) ...1  ***አንዳንዴ***(ከ3-10 ግዜ በወር ዉስጥ)......2  ***ብዙጊዜ*** (ከ10 ግዜ በላይ በወር ዉስጥ)...3 | | | | | | | | | | | |  | | | |
| 205 | | ባለፈው አንድ ወር እርስዎ ወይንም ሌላ የቤተሰብዎ አባል በአቅም ማጣት ምክንያት በየቀኑ ተመሳሳይ ምግብ ተመግበው ያውቀሉ? ለምሳሌ ሁሌ ጎመን ብቻ | | | | አዎ .............................................1  የለም ........................................ 0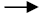 | | | | | | | | | | | | 207 | | | |
| 206 | | ባለፈው አንድ ወር ውስጥ ይህ ችግር ምን ያህል ግዜ ተከስቷል? | | | | ***አልፎ አልፎ***(1- 2 ግዜ በወር ዉስጥ) ...1  ***አንዳንዴ***(ከ3-10 ግዜ በወር ዉስጥ)......2  ***ብዙጊዜ*** (ከ10 ግዜ በላይ በወር ዉስጥ)...3 | | | | | | | | | | | |  | | | |
| 207 | | ባለፈው አንድ ወር እርስዎ ወይንም ሌላ የቤተሰብዎ አባል በአቅም ማነስ ምክንያ በአካባቢው ማህበረሰብ ዘንድ ያልተለመዱ (የማይበሉ) ምግቦችን ተመግበው ያውቀሉ? በችግር ግዜ ብቻ የሚበላ | | | | አዎ .............................................1  የለም ...........................................0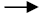 | | | | | | | | | | | | 209 | | | |
| 208 | | ባለፈው አንድ ወር ውስጥ ይህ ችግር ምን ያህል ግዜ ተከስቷል? | | | | ***አልፎ አልፎ***(1- 2 ግዜ በወር ዉስጥ) ...1  ***አንዳንዴ***(ከ3-10 ግዜ በወር ዉስጥ)......2  ***ብዙጊዜ*** (ከ10 ግዜ በላይ በወር ዉስጥ)...3 | | | | | | | | | | | |  | | | |
| 209 | | ባለፈው አንድ ወር እርስዎ ወይንም ሌላ የቤተሰብዎ አባል በቤትዎ በቂ ምግብ ባለመኖሩ ምክንያት ከሚያስፈልግዎ ምግብ መጠን ያነሰ ተመግበዋል? | | | | አዎ ............ 1 የለም ............ 0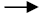 | | | | | | | | | | | | 211 | | | |
| 210 | | ባለፈው አንድ ወር ውስጥ ይህ ችግር ምን ያህል ግዜ ተከስቷል? | | | | ***አልፎ አልፎ***(1- 2 ግዜ በወር ዉስጥ) ...1  ***አንዳንዴ***(ከ3-10 ግዜ በወር ዉስጥ)......2  ***ብዙጊዜ*** (ከ10 ግዜ በላይ በወር ዉስጥ)...3 | | | | | | | | | | | |  | | | |
| 211 | | ባለፈው አንድ ወር እርስዎ ወይንም ሌላ የቤተሰብዎ አባል በቤትዎ በቂ ምግብ ባለመኖሩ ምክንያት በቀን ውስጥ ከተለመደው ያነሰ ግዜ ምግብ ተመግበው ያውቃሉ? (ለምሳሌ በቀን ሶስት ግዜ ይመገቡ ከነበረ.፤ከሶስት ግዜ ያነሰ) | | | | አዎ ..............................................1  የለም ...........................................0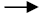 | | | | | | | | | | | | 213 | | | |
| 212 | | ባለፈው አንድ ወር ውስጥ ይህ ችግር ምን ያህል ግዜ ተከስቷል? | | | | *አልፎ አልፎ* (1- 2 ግዜ በወር ዉስጥ).....1  *አንዳንዴ* (ከ3 - 10 ግዜ በወር ዉስጥ).....2  *ብዙጊዜ* (ከ10 ግዜ በላይ በወር ዉስጥ)........3 | | | | | | | | | | | |  | | | |
| 213 | | ባለፈው አንድ ወር የቤተሰብዎ አባል በቂ ምግብ ባለመኖሩ ምክንያት በቤት ውስጥ የሚላስ የሚቀመስ ጠፍቶ ያዉቃል? | | | | አዎ .............................................. 1  የለም ..........................................0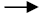 | | | | | | | | | | | | 215 | | | |
| 214 | | ባለፈው አንድ ወር ውስጥ ይህ ችግር ምን ያህል ግዜ ተከስቷል? | | | | *አልፎ አልፎ* (1-2 ግዜ በወር ዉስጥ) ....1  *አንዳንዴ* (ከ3-10 ግዜ በወር ዉስጥ).......2  *ብዙጊዜ* (ከ10 ግዜ በላይ በወር ዉስጥ)......3 | | | | | | | | | | | |  | | | |
| 215 | | ባለፈው አንድ ወር እርስዎ ወይንም ሌላ የቤተሰብዎ አባል በቂ ምግብ ባለመኖሩ ምክንያት የተነሳ ሳትበሉ ያደራቹበት ቀን አለ? | | | | አዎ ...........1 የለም ................. 0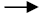 | | | | | | | | | | | | 217 | | | |
| 216 | | ባለፈው አንድ ወር ውስጥ ይህ ችግር ምን ያህል ግዜ ተከስቷል? | | | | *አልፎ አልፎ* (1-2 ግዜ በወር ዉስጥ) ....1  *አንዳንዴ* (ከ3-10 ግዜ በወር ዉስጥ).......2  *ብዙጊዜ* (ከ10 ግዜ በላይ በወር ዉስጥ).....3 | | | | | | | | | | | |  | | | |
| 217 | | ባለፈው አንድ ወር እርስዎ ወይንም ሌላ የቤተሰብዎ አባል በቂ ምግብ ባለመኖሩ ምክንያት የተነሳ ሳትበሉ ውለው፤አድረው የውቃሉ? | | | | አዎ -- 1 የለም -- 0 | | | | | | | | | | | |  | | | |
| 218 | | ባለፈው አንድ ወር ውስጥ ይህ ችግር ምን ያህል ግዜ ተከስቷል? | | | | *አልፎ አልፎ* (1-2 ግዜ በወር ዉስጥ) ....1  *አንዳንዴ* (ከ3-10 ግዜ በወር ዉስጥ).......2  *ብዙጊዜ* (ከ10 ግዜ በላይ በወር ዉስጥ)............3 | | | | | | | | | | | |  | | | |
| **ክፍል ሦስት፡ የቤተሰብ እና የህፃኑን የአመጋገብ ስብጥር በተመለከተ**  **ሀ. የልጁን/ቷን የምግብ ድግግሞሽ ለማወቅ የተዘጋጀ መጠይቅ** | | | | | | | | | | | | | | | | | | | | | |
|  | | የምግብ ዝርዝር | | | | | የ 30 ቀናት የአመጋገብ ድግግሞሽ | | | | | | | | | | | | | | |
|  | | እባክዎ ከተዘረዘሩት የምግብ ዝርዝሮች በአማካኝ ምን ያህል እንደተጠቀመ ይምረጡ | | | | | **በወር** | | | | **በሳምንት** | | | | **በቀን** | | | | | | |
|  |  |  |  |  |  |  | **ጭራሽ አልተጠ-ቀምኩም** | **ኣንድ ጊዜ** | | 2–3 ጊዜ | 1–2 ጊዜ | 3–4ጊዜ | | 5-6 ጊዜ | 1–2 ጊዜ | 2- 3 ጊዜ | 4-5 ጊዜ | | | | >6 ጊዜ |
| 301 | | **ከእህል ዘር የተዘጋጁ ምግቦች** | | | | |  | | | | | | | | | | | | | | |
|  |  | ከእህል ዘር የተዘጋጁ ምግቦች እንደ እንጀራ፣ዳቦ፣ መኮሮኒ፣ ገንፎ፣ፍርፍር፣ አጥሚት፣ፓስታ፤ሩዝ፤ወይም ከአጃ፤ በቆሎ፤ ገብስ፤ ስንዴ፤ ማሽላ ወይም ከሌሎች የእህል ዘር የተሰራ ምግብ | | | | |  |  | |  |  |  | |  |  |  |  | | | |  |
| 302 | | **ስራቸው የሚበሉ ምግቦች** | | | | |  |  | |  |  |  | |  |  |  |  | | | |  |
|  |  | ድንች፤ስኳርድንች፤ቀይስር፤ወይም ሌሎች ስራቸው የሚበሉ | | | | |  |  | |  |  |  | |  |  |  |  | | | |  |
| 303 | | **የአትክልት ምግቦች** | | | | |  |  | |  |  |  | |  |  |  |  | | | |  |
|  |  | እንደ ሙዝ፣ጎመን፤ሰላጣ፤ካሮት፤ቆስጣ፣ቃሪያ፣ ቲማቲም፣ በርበሬ፣ሽንኩርት፣ሌሎች ደማቅ አረንጓዴ ቅጠል ያላቸው አትክልቶች | | | | |  |  | |  |  |  | |  |  |  |  | | | |  |
| 304 | | **ፍራፍሬ** | | | | |  |  | |  |  |  | |  |  |  |  | | | |  |
|  |  | የፍራፍሬ አጠቃቀም እንደ ብረቱካን፣ ማነጎ ፣ አቦካዶ፣ ሎሚ፣ መንደሪን፣ አናናስ፣ዘይቱን፣አፕል፣ኮክ ወ.ዘ.ተ | | | | |  |  | |  |  |  | |  |  |  |  | | | |  |
|  |  | የፍራፍሬ ጭማቂ አጠቃቀም እንደ ማንጎ፣ አቮካዶ፣ ፐሪጋት፣ ራኒ ወ.ዘ.ተ | | | | |  |  | |  |  |  | |  |  |  |  | | | |  |
| 305 | | **ስጋ ነክ ምግቦች** | | | | |  |  | |  |  |  | |  |  |  |  | | | |  |
|  |  | ስጋ ነክ ምግቦች እንደ ጥብስ፣ ቀይ ወጥ፣ ስጋ ፍርፍር፣ ወ.ዘ.ተ. ከላም፣ የበሬ፣ የበግ፣ የፍየል፣ የዶሮ ስጋ የተዘጋጁ ምግቦች | | | | |  |  | |  |  |  | |  |  |  |  | | | |  |
| 306 | | እንቁላል ነክ ምግቦች | | | | |  |  | |  |  |  | |  |  |  |  | | | |  |
|  |  | ከእንቁላል የተሰሩ ምግቦች እንደ እንቁላል ጥብስ፣ ሳንዱች ፣ | | | | |  |  | |  |  |  | |  |  |  |  | | | |  |
|  | | እባክዎ ከተዘረዘሩት የምግብ ዝርዝሮች በአማካኝ ምን ያህል እንደተጠቀመ ይምረጡ | | | | | **በወር** | | | | **በሳምንት** | | | | **በቀን** | | | | | | |
|  |  |  |  |  |  |  | **አልተጠ-ቀምኩም** | **ኣንድ ጊዜ** | | 2–3 ጊዜ | 1–2 ጊዜ | 3–4ጊዜ | | 5-6 ጊዜ | 1–2 ጊዜ | 2- 3 ጊዜ | 4-5 ጊዜ | | | | >6 ጊዜ |
| 307 | | ዓሣና ሌሎች የባህር ምግቦች | | | | |  |  | |  |  |  | |  |  |  |  | | | |  |
| 308 | | **ከጥራጥሬ የተዘጋጁ ምግቦች** | | | | |  |  | |  |  |  | |  |  |  |  | | | |  |
|  |  | ባቄላ፤አተር፤አኩሪ አተር፤ቦለቄ፤ ኑግ፤ሰሊጥ፤ለውዝ ወይንም ከሌሎች ጠራጥሬዎች የተሰራ ምግብ | | | | |  |  | |  |  |  | |  |  |  |  | | | |  |
| 309 | | **ወተት እና የወተት ተዋጽዖ ምግቦች** | | | | |  |  | |  |  |  | |  |  |  |  | | | |  |
|  |  | ወተት እና የወተት ተዋጽዖ እንደ አይብ ፣ እርጎ ፣ ወተት ወ.ዘ.ተ .ልብ ይበሉ ቅቤን አይጨምርም | | | | |  |  | |  |  |  | |  |  |  |  | | | |  |
| 310 | | **በዘይት የተጠበሱ ምግቦች** | | | | |  |  | |  |  |  | |  |  |  |  | | | |  |
|  |  | ማንኛም ዘይት ወይንም በቅቤ የተሰራ ምግብ እንደ ችብስ፣ ቦምቦሊኖ፣ ሳመቡሳ፣ ዶናት፣ሳንዱች፣ በርገር | | | | |  |  | |  |  |  | |  |  |  |  | | | |  |
| 311 | | ጣፋጭ ምግቦችስኳር፤ማር | | | | |  |  | |  |  |  | |  |  |  |  | | | |  |
|  |  | ጣፋጭ ምግቦች እንደ ስኳር፣ማር ስኳር ድንች፣ ሸንኮር አገዳ | | | | |  |  | |  |  |  | |  |  |  |  | | | |  |
|  |  | ቸኮሌት፣ከረሜላ፣አምባሻ/ሳምቡሳ፣ኩኪስ ወ.ዘ.ተ | | | | |  |  | |  |  |  | |  |  |  |  | | | |  |
| 312 | | **መጠጦች** | | | | |  |  | |  |  |  | |  |  |  |  | | | |  |
|  |  | ለስላሳ መጠጦች (ኮካ፣ ሚሪንዳ፣ ፔፕሲ፣ ሰቭን አብ፣ ወ.ዘ.ተ) | | | | |  |  | |  |  |  | |  |  |  |  | | | |  |
|  |  | ሻይ (የቅጠል ሻይ፣ ማንጎ ሻይ፣ አናናስ ሻይ፣ ወ.ዘ.ተ) | | | | |  |  | |  |  |  | |  |  |  |  | | | |  |
|  |  | የቡና አጠቃቀም | | | | |  |  | |  |  |  | |  |  |  |  | | | |  |
|  |  | አልኮል መጠጦች እንደ ጠላ፣ ወይን፣ቢራ፣ወ.ዘ.ተ | | | | |  |  | |  |  |  | |  |  |  |  | | | |  |
| **ክፍል አራት: ሀ. የህፃናቱን ማህበረሰባዊ መገለጫ በተመለከተ** | | | | | | | | | | | | | | | | | | | | | |
| 401 | | ልጁ/ቷ ፆታው/ዋ ምንድን ነው? | | | ወንድ………1 ሴት………2 | | | | | | | | | | | | | | |  | |
| 402 | | ልጁ/ቷ በየትኛው ወርና አመተ ምህረት ነው የተወለደው/ችው? | | | ወር____አላውቅም………98 አመት____አላውቅም………98 | | | | | | | | | | | | | | |  | |
| 403 | | ልጁ/ቷ ስንት አመት ሆነው/ሆናት? | | | እድሜ በተተናቀቀ ወር ሲቆጠር------------- | | | | | | | | | | | | | | |  | |
| 404 | | ልጁ/ቷ በአሁን ሰአት ስንተኛ ክፍል እየተማረ/ች ነው? | | | ያለፈውን ያጠናቀቀችውን/ውን ክፍል……… | | | | | | | | | | | | | | |  | |
| **ለ. የህፃናቱን የጤና ሁኔታ በተመለከ** | | | | | | | | | | | | | | | | | | | | | |
| 405 | | ባለፉት ሰባት ቀናት ውስጥ ልጁ/ቷ የብረት(አይረን) እንክብል ወስዶ/ዳ ያውቃል/ታውቃለች? | | አዎ----------1 ወስዶ/ዳ አታውቅም--------2  እኔ አላውቅም------------98 | | | | | | | | | | | | | | | |  | |
| 406 | | ባለፉት ስድስት ወራት ውስጥ ልጁ/ቷ ቫይታሚን ኤ ወስዶ/ዳ ያውቃል/ታውቃለች? | | አዎ----------1 ወስዶ/ዳ አታውቅም--------2  እኔ አላውቅም------------98 | | | | | | | | | | | | | | | |  | |
| 407 | | ባለፉት ስድስት ወራት ውስጥ ልጁ/ቷ የአንጀት ትላትል መድሃኒት ወስዶ/ዳ ያውቃል/ታውቃለች? | | አዎ----------1 ወስዶ/ዳ አታውቅም--------2  እኔ አላውቅም------------98 | | | | | | | | | | | | | | | |  | |
| 408 | | ባለፉት ሁለት ሳምንታት ውስጥ ልጁ/ቷ በማንኛውም ህመም ታመው ያውቃሉ? | | አዎን……1 አልታመመም/ችም……… 2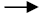  አላወኩም…………….98 | | | | | | | | | | | | | | | | 413 | |
| 409 | | ባለፉት ሁለት ሳምንታት ውስጥ ልጁ/ቷ ትኩሳት ኖሮባት/በት ያውቃል/ታውቃለች? | | አዎን…1 የለም……0 አላወኩም…98 | | | | | | | | | | | | | | | |  | |
| 410 | | ባለፉት ሁለት ሳምንታት ውስጥ ልጁ/ቷ ሳል ኖሮባት/በት ያውቃል/ታውቃለች? | | አዎን…1 የለም……0 አላወኩም…98 | | | | | | | | | | | | | | | |  | |
| 411 | | ባለፉት ሁለት ሳምንታት ውስጥ ልጁ/ቷ ተቅማጥ ኖሮባት/በት ያውቃል/ታውቃለች? | | አዎን…1 የለም……0 አላወኩም…98 | | | | | | | | | | | | | | | |  | |
| 412 | | ከሰገራው ጋር ደም ይታይ ነበር? | | አዎን…1 የለም……0 አላወኩም…98 | | | | | | | | | | | | | | | |  | |
| 413 | | በቤቱ ውስጥ የወባ መከላከያ አጎበር አለ? | | አዎን…1 የለም……0 አላወኩም…98 | | | | | | | | | | | | | | | |  | |
| 414 | | ምን ያህል ግዜ ሆኖታል አጎበሩን ካገኛችሁ? | | ወር በፊት…1 አመት በፊት--2 አላወኩም--98 | | | | | | | | | | | | | | | |  | |
| 415 | | ትላንትና ሌሊት ልጁ/ቷ አጎበር ተጠቅመዋል? | | አዎን…1 የለም……0 አላወኩም…98 | | | | | | | | | | | | | | | |  | |

**የ 24 ሰዓትን የምግብ ስብጥር በተመለከተ የተዘጋጀ መጠይቅ**

| ባለፉት **24** ሰዓት ዉስጥ ልጅዎ የተመገባቸውን ምግቦች ኣስመልክቶ ለመጠየቅ ቀረቡ ጥቄዎች | | | | | | | | |
| --- | --- | --- | --- | --- | --- | --- | --- | --- |
| ተ.ቁ | ምግቡ የተበላበት ጊዜ | | | ምግቡ አይነት | | የምግቦቹ ዝርዝር | | |
| 1 | ጠዋት ቁርስ | | |  | |  | | |
| 2 | ከቁርስ በኋላ(መቆያ) | | |  | |  | | |
| 3 | ቀን ምሳ | | |  | |  | | |
| 4 | ከምሳ በኋላ (መክሰስ) | | |  | |  | | |
| 5 | ማታ እራት | | |  | |  | | |
| 6 | ከእራት በኋላ(መቆያ) | | |  | |  | | |
| **2**. የምግቦቹን ዉህዶች ገለፃ ፎርም | | | | | | | | |
| ኣሁን ደግሞ ከላይ የዘረዘሩልኝ ምግቦች ዉህድቹ እና የዉህድቹን መጠን፣ አይነት እና ልጅዎ ምንያህል አንደበላ**/**ጠጣች በዝርዝር ይነግሩኛል፡፡ | | | | | | | | |
|  | | የምግቡ/መጠጡ ዝርዝር እና አይነቱ | | | የምግቡ/መጠጡ ገለፃ: የዉህድቹ ዝርዝር | | | መጠናቸዉ |
| 1 | |  | | |  | | |  |
| 2 | |  | | |  | | |  |
| 3 | |  | | |  | | |  |
| 4 | |  | | |  | | |  |
| 5 | |  | | |  | | |  |
| 6 | |  | | |  | | |  |
| 7 | |  | | |  | | |  |
| 8 | |  | | |  | | |  |
| **ተጠያቂዎቹ መመልሳቸውን ካቆሙ(ከጨረሱ) በኋላ እያንዳንዱን የተዘረዘሩትን ምግቦች በተዘረዘረው የምግብ ምድብ በጥንቃቄ ስቀምጥ/አስቀምጪ** | | | | | | | | |
| 1 | **ከእህል ዘር የተዘጋጁ ምግቦች** | | ከእህል ዘር የተዘጋጁ ምግቦች እንደ እንጀራ፣ዳቦ፣ መኮሮኒ፣ ገንፎ፣ፍርፍር፣ አጥሚት፣ፓስታ፤ሩዝ፤ወይም ከአጃ፤ በቆሎ፤ ገብስ፤ ስንዴ፤ ማሽላ ወይም ከሌሎች የእህል ዘር የተሰራ ምግብ | | | | አዎ---1 የለም---2 | |
| 2 | **ስራቸው የሚበሉ ምግቦች** | | ድንች፤ስኳርድንች፤ቀይስር፤ወይም ሌሎች ስራቸው የሚበሉ | | | |  | |
| 3 | **የአትክልት ምግቦች** | | እንደ ሙዝ፣ጎመን፤ሰላጣ፤ካሮት፤ቆስጣ፣ቃሪያ፣ ቲማቲም፣ በርበሬ፣ሽንኩርት፣ሌሎች ደማቅ አረንጓዴ ቅጠል ያላቸው አትክልቶች | | | |  | |
| 4 | **ፍራፍሬ** | | የፍራፍሬ አጠቃቀም እንደ ብረቱካን፣ ማነጎ ፣ አቦካዶ፣ ሎሚ፣ መንደሪን፣ አናናስ፣ዘይቱን፣አፕል፣ኮክ ወ.ዘ.ተ | | | |  | |
|  |  |  | የፍራፍሬ ጭማቂ አጠቃቀም እንደ ማንጎ፣ አቮካዶ፣ ፐሪጋት፣ ራኒ | | | |  | |
| 5 | **ስጋ ነክ ምግቦች** | | ስጋ ነክ ምግቦች እንደ ጥብስ፣ ቀይ ወጥ፣ ስጋ ፍርፍር፣ ወ.ዘ.ተ. ከላም፣ የበሬ፣ የበግ፣ የፍየል፣ የዶሮ ስጋ የተዘጋጁ ምግቦች | | | |  | |
| 6 | እንቁላል ነክ ምግቦች | | ከእንቁላል የተሰሩ ምግቦች እንደ እንቁላል ጥብስ፣ ሳንዱች ፣ ፍርፍር፣ቅቅል፣ የእንቁላል ወጥ፣ ወ.ዘ.ተ… | | | |  | |
| 7 | **ዓሳ ምግቦች** | | ዓሣና ሌሎች የባህር ምግቦች | | | |  | |
| 8 | **ከጥራጥሬ የተዘጋጁ ምግቦች** | | ባቄላ፤አተር፤አኩሪ አተር፤ቦለቄ፤ ኑግ፤ሰሊጥ፤ለውዝ ወይንም ከሌሎች ጠራጥሬዎች የተሰራ ምግብ | | | |  | |
| 9 | **ወተት እና የወተት ተዋጽዖ ምግቦች** | | ወተት እና የወተት ተዋጽዖ እንደ አይብ ፣ እርጎ ፣ ወተት ወ.ዘ.ተ .ልብ ይበሉ ቅቤን አይጨምርም | | | |  | |
| 10 | **በዘይት የተጠበሱ ምግቦች** | | ማንኛም ዘይት ወይንም በቅቤ የተሰራ ምግብ እንደ ችብስ፣ ቦምቦሊኖ፣ ሳመቡሳ፣ ዶናት፣ሳንዱች፣ በርገር | | | |  | |
| 11 | ጣፋጭ ምግቦችስኳር፤ማር | | ጣፋጭ ምግቦች እንደ ስኳር፣ማር ስኳር ድንች፣ ሸንኮር አገዳ | | | |  | |
|  |  |  | ቸኮሌት፣ከረሜላ፣አምባሻ/ሳምቡሳ፣ኩኪስ ወ.ዘ.ተ | | | |  | |
| 12 | **መጠጦች** | | ለስላሳ መጠጦች (ኮካ፣ ሚሪንዳ፣ ፔፕሲ፣ ሰቭን አብ፣ ወ.ዘ.ተ) | | | |  | |
|  |  |  | ሻይ (የቅጠል ሻይ፣ ማንጎ ሻይ፣ አናናስ ሻይ፣ ወ.ዘ.ተ) | | | |  | |
|  |  |  | የቡና አጠቃቀም | | | |  | |

**ውድ ጊዜዎትን ሰውተው ላደረጉልኝ ትብብር ከልብ አመሰግና!!**
